# Illustrations of the Sedative and Febrifuge Powers of Emetic Tartar

**Published:** 1824-05

**Authors:** William Balfour

**Affiliations:** the University of Edinburgh.


ART. V.—*Illustrations of the Sedative and Febrifuge Powers of Emetic Tartar.* By WILLIAM BALFOUR, M.D. of the University of Edinburgh.

Ne quid falsi dicere audeam, ne quid veri non audeam.

[Continued from page 283.]

No impartial person, I presume, will hesitate to admit my claim to originality, in what I have advanced concerning the sedative virtues of tartrite of antimony and potass; but the evidence I can adduce amounts to demonstration: so that it would be as easy to deny that the opposite sides of a square are equal, as to deny that I have made important discoveries with regard to the sedative powers of emetic tartar.

But, before proceeding to adduce my evidence, I must premise, what no sane person will attempt to controvert, that an improvement in the mode of exhibiting a medicine may be equivalent to the discovery of a new remedy; and may justly be considered as important a discovery as if the remedy had never been heard of before. What signifies it that the name of a medicine is known, if practitioners are ignorant of the circumstances, and of the doses, in which it proves most beneficial?

In the first place, then, I would ask those gentlemen, who think that, because a medicine has long had a place in the Pharmacopœias, its medicinal powers must be perfectly and thoroughly known,—Where, and by whom, any facts are recorded, equivalent to those I have brought forward? When, and by whom, was ever pneumonia, ophthalmia, nephritis,

* See *Illustrations of the Sedative and Febrifuge Powers of Emetic Tartar*, second edition, 1819.

croup, asthma, hæmoptysis, apoplexy, &c. cured by emetic tartar, without blood-letting? Are there, in the records of medicine, two such cases as those of Master Ormston and Mrs. Lawson? What author ever made use of the language concerning the medicinal powers of emetic tartar, that I have employed? Till satisfactory answers are made to these queries, *which never can be done*, the irresistible conclusion is, that my observations on the subject are original.

In the second place, if emetic tartar had been "known before" as well as it is now, such knowledge must have had a proportionate influence on practice. But, if it is inquired what that influence is, it will be found to be nothing. Blood-letting is practised, at this day, to all the destructive extent to which it ever was carried; not by obscure, illiterate members of the profession only, but by the most learned and eminent. Charity then compels us to conclude, that they who carry depletion such dangerous lengths are *still* ignorant of the qualities which I have discovered in emetic tartar. No man in his senses would carry any particular remedy the length of rendering it equivocal whether the patient is in most danger from the doctor or from the disease, were he convinced he had another remedy, little less efficacious and infinitely less dangerous, in his power. How, then, could those qualities I have discovered in emetic tartar "be known before," which are not yet acknowledged?

In the third place, it is evident, from the latest and most approved practice in medicine and surgery, that the beneficial effects of antimonial medicines in inflammatory complaints are universally ascribed to the *nausea* they produce. In proof of this, I appeal to Thomas's "Practice of Physic," and Cooper's "Dictionary of Surgery," avowedly books of reference, as containing the opinions and practice of the most eminent of the profession, to this very day and hour.

"Emetic medicines," says Dr. Thomas, "and particularly antimonials, given in small *nauseating* doses, (the sixth, or at most the fourth, of a grain every two or three hours,) have likewise a similar power (to neutral salts) of determining the circulation to the surface of the body. From the uncertainty with which Dr. James's powder and the pulvis antimonialis act (in producing nausea), the tartarized antimony may be considered as preferable in many cases."*

From this it is evident Dr. Thomas does not regard emetic tartar as a remedy pre-eminent in the cure of inflammation. He generally employs it in combination with, but certainly not in preference to, the common neutral salts, and merely as pos-

* Dr. THOMAS's *Practice of Physic*, seventh edition, 1821; page 36.

sessing, in common with them, the power of determining to the surface. Accordingly, when intended to lower the action of the system, he uniformly prescribes it in small nauseating doses; a certain proof that, in such cases, he considers its medicinal, as altogether dependent on its nauseating, powers.

"Nauseating medicines," says Mr. Cooper, "employed after bleeding, has been practised once or twice, are often productive of considerable benefit. The tartrite of antimony (emetic tartar,) is the one on which practitioners place the greatest reliance; and it is to be prescribed for the purpose of exciting nausea, as directed below:" that is, the fourth of a grain is to be given every sixth hour. "The softest diaphoretics are the citrate of potass, the acetate of ammonia, the tartrite of antimony, and James's powder. The two latter, from their effects in producing nausea and weakening the pulse, are sometimes most efficacious, as already stated."*

Thus it is manifest that these two eminent authors had not, when they last wrote on the subject, the most distant idea of emetic tartar being capable of diminishing the action of the system, otherwise than by previously inducing nausea. Nay, they expressly prefer it to other sudorifics, on no other ground than its more certainly and quickly producing nausea.

But it is ascertained by experience that doses of emetic tartar, which produce both nausea and vomiting at first, do not *continue* to do so, if administered at stated intervals. The stomach accommodates itself to the action of emetic tartar, as well as to that of other substances which powerfully affect it at first. If, therefore, the principle on which our authors exhibit the medicine is just, their practice cannot but be absurd.

If, to give effect to the medicine, nausea must be produced, every succeeding dose must also, to a certain extent, be increased. Of the necessity of this, however, our authors give not the slightest hint. Now, is it to be believed for a moment that they practise as they write? They cannot. No man can; because patients will not submit to the distress of perpetual nausea.

But why not employ emetic tartar till "after bleeding has been practised once or twice?"† By giving a grain of the medicine, in Mrs. Lawson's case, *before bleeding was practised at all*, and half a grain at intervals of half an hour and an hour, the necessity of bleeding a second time was prevented.

Let us now hear what those of the Edinburgh school have to say to emetic tartar as a remedy for inflammation; and for this purpose I select Dr. Thomson's "Lectures on Inflammation."

---

* Mr. Samuel Cooper's *Dictionary of Surgery*, third edition, 1818; page 634.
† Cooper.

I won't leave you a hole to creep out at.

There the medicine is adverted to under the head of diaphoretics; and merely as diaphoretic, emetic, or purgative.—"These antimonial diaphoretics," says the Professor, "from their effects in producing *sickness* and weakening the pulse, may in some cases have a superior efficacy." Again, "In the inflammatory affections, accompanied with bilious or gastric symptoms, these substances (antimonial diaphoretics) may be employed in small and repeated doses, so as to produce also an emetic or purgative effect. The late Mr. Dease, of Dublin, and M. Desault, used to give a weak solution of emetic tartar for the cure of erysipelas. I have often imitated their practice in this particular, and with the most beneficial effects, though the medicine, during its employment, often occasioned a considerable and distressing degree of sickness."*

Here there is not the most distant allusion to the medicine in question, as possessed of any other than evacuant and nauseating powers: nay, the use of it is virtually proscribed, "as often occasioning a distressing degree of *sickness*." Will it be pretended, then, that Dr. Thomson, when he published his "Lectures on Inflammation," knew that emetic tartar, when given at stated intervals, even in doses which at first produce both nausea and vomiting, ceases to produce either of these effects; and that it continues to lower the action of the system, without producing any sensible evacuation whatever? Yet Dr. Thomson is a physician, in point of talent and information, second to none of his day.

On the whole, no proof can be deduced, from the best and latest writers, that the medicinal powers of emetic tartar are better understood at the present day, than they were by Dr. Cullen, who uniformly prescribed it in nauseating doses; whereby he subjected his patients to unnecessary distress, and brought the medicine itself into unmerited disrepute.

I was called to T—, aged twenty-seven, on the 19th April, 1820. He had been confined precisely five weeks, by pneumonia and general rheumatism combined. In the course of that time, he had been bled seven or eight times,—altogether, to upwards of a hundred ounces: inflammatory action, however, was by no means subdued. He complained of pain and oppression in the chest, which precluded him making a full inspiration. These, it was believed, were owing to a rheumatic affection of the external muscles of the thorax; a mistake, of which any one might very easily have convinced himself. Rheumatism was now confined almost exclusively to the arms, (unless the internal affection was also rheumatic,) which were

* *Lectures on Inflammation*, 1813; page 172.

acutely painful, swollen at the joints, and immovable. Great general debility; much thirst; pulse 120, full and strong.

The only medicine I found the patient taking was stramonium, which, he said, uniformly made him hot, restless, and thirsty; effects which might have been anticipated.

I was given to understand that I was called in on account of the rheumatic affection of the arms, which, in spite of all the bleeding that had been practised, was getting worse daily. I informed the patient that, whatever his present feelings might be, or however apprehensive of long confinement, rheumatism in the arms was the least of his complaints; that the high action of the system, and the internal affection of the chest, impeding respiration, demanded primary attention; and that, when these were subdued, the rheumatic affection of the arms would be easily removed.

I commenced my treatment of this case by applying bandages to the arms, which the patient could not lift from the bed-clothes, and by exhibiting emetic tartar internally. In twenty-four hours, the patient recovered the use of his hands and arms in a great degree; and the pulse came down from 120 to 94, in the same short space of time. In these circumstances, he thought proper to rise and put on his clothes. The consequence was, his pulse, on the evening of the second day of my attendance, rose to 116, and was strong and hard. I remonstrated with the patient on his conduct in pretty strong terms, when he promised, and afterwards gave, punctual obedience. The second dose of the medicine having occasioned nausea and slight vomiting, it was afterwards taken with unnecessary caution. This proceeded partly, however, from misapprehension of the directions given. Accordingly, two days more elapsed before much impression was again made on the action of the system. I insisted on a more liberal use of the medicine, assuring the patient he had nothing to fear, but every thing to hope, from it. Henceforth it was taken in increased doses, and frequently; but never again occasioned vomiting, nausea, or any inconvenience whatever. In eight days inclusive from the time I was called in, fourteen grains of the medicine were taken, and every symptom of the disease had disappeared.

In their blind and unreasonable opposition to the practice I have introduced in rheumatism, some medical practitioners have triumphantly asked, how a part in a state of inflammation could be touched? how percussion could be applied where fever is present? In reply, I have to state that, in the present case, I handled the parts affected with rheumatism with the most soothing effects; and that I applied percussion to the shoulders and back when the pulse was 120, not only without injury, but the pulse came down during the operation, sometimes six, and at other times ten, strokes in a minute. I repeatedly called the attention of the patient to this circumstance. I have observed the same thing in other patients; and I think the effect may be easily accounted for, on the principle of percussion equalizing the circulation and nervous power.

It would be difficult, I think, to conceive a more satisfactory illustration of the superiority of the practice I have introduced in the treatment of inflammatory complaints, to that universally followed at the present day, than what this case affords. The patient had been confined five weeks, was bled seven or eight times, and reduced to a state of great debility; still none of the urgent symptoms were removed. The inflammatory action of the system remained unsubdued; the rheumatic affection in the extremities having increased, the pain in the chest still impeding respiration, and the pulse remaining as high as 120, full and strong; circumstances indicating blood-letting as imperatively as at the beginning of the disease. No sooner was emetic tartar administered, however, than the pulse came down from 120 to 94; and, but for the imprudence of the patient, the cure would have been completed almost immediately. I am, therefore, warranted to infer that, had this gigantic medicine been exhibited from the beginning in the quantity, and with the frequency, that I employed it, the patient would not have been confined a week.

But why was not emetic tartar administered at all by the gentlemen who preceded me? The only answer that can be given is, they were ignorant of its powers: nay, that they refused to believe it possessed of the virtues which I have ascribed to it. Had it been otherwise, it is impossible they could have bled their patient seven or eight times, to the utter neglect of a medicine which would have saved three-fourths of such depletion, and of the sufferings of the patient.

"But there are some affections," Mr. Cooper says, "in which they (nauseating medicines) cannot be used, such as inflammation of the stomach and intestines."* This proposition I shall neither affirm nor deny, but content myself with laying before the reader the three following cases; requesting him, at the same time, to look back to Mrs. Lawson's second illness, in which, though there was a very strong tendency to inflammatory action in both stomach and bowels, emetic tartar produced the most benign effects.

Margaret Ramsay, aged fifty-three, became my patient on the 31st May, 1820, for rheumatism in her ankles and right arm. She had been unable to walk, except with great pain, for many weeks; during which time she had medical advice, without deriving any benefit from it. In less than a week I suc-

* *Dictionary of Surgery, loco citato.*

ceeded in curing these affections, although her arm was so pained that she could not lift it from the bed.

Notwithstanding my injunctions to avoid cold and damp, she no sooner found her pains removed than she applied herself to the washing and dressing of clothes. I had but left off attending her, therefore, when I was recalled, and found her labouring under fixed pungent pain immediately under the left breast, accompanied with incessant cough; pulse 100, and hard; I opened a vein, but got the length of half a cup only when she fainted, without giving any warning. I kept my thumb on the orifice of the wound, with the intention of drawing off the requisite quantity of blood when she recovered; but did not succeed in getting any more. I gave her a small dose of antimonial wine, and assured her she would soon feel the benefit of the fainting-fit she had experienced. The cough and pain left her in a quarter of an hour, and never returned. She was now removed, being a servant, to another house, where she had better accommodation; and, though the local affection was removed, fever continued for some days. This patient refused to take medicine; at least, in such quantity as would effect any change in the action of the system. At length, without any apparent cause, fever increased; unaccompanied, however, with any pain in the chest, or any where else. She was now prevailed upon to take emetic tartar in increased, though not nauseating, doses; and the action of the system was thereby reduced, in the course of one day, from 110 to 80 strokes of the pulse in a minute, accompanied with a breathing sweat. This took place on the 12th of June. I told the patient the cure was complete, provided she took only ordinary care of herself. Unfortunately, however, in the state of perspiration just described, she took repeated draughts of cold water and small beer, and was immediately seized with pain in the stomach, tending to the abdomen. Emetic tartar, combined with the saline julep, administered so as to effervesce in the stomach, was resumed; and the pain very soon left the stomach, but became fixed and acute in the abdomen.

On the 13th, I prevailed on the patient to suffer me to bleed her again; which I did twice that day, both bleedings amounting to only twenty-five ounces. So ill did she stand bleeding, however, that I did not expect she would have recovered from the collapse that ensued. Inflammatory action was, of course, suspended; but returned, as re-action was restored, in all its former violence. To blistering she would not submit, and declared her willingness to die rather than be bled again. I had no resource left, therefore, but in emetic tartar and the saline julep. These the patient took freely, as her only remaining chance, and with such success that pain was almost entirely removed on the evening of the 15th: the pulse had come down to 80, was full and soft; and she had a warm fluid sweat over the whole body. Dr. Abercrombie also saw the patient in these circumstances, and admitted he considered the disease as subdued. A relapse took place in the night, however; and, on the morning of the 18th, death closed the scene.

Here, then, is a case of enteritis, in which emetic tartar was employed with the very best effects, without ever occasioning either nausea or vomiting; and, if the medicine had been taken as liberally from the beginning of even the affection of the stomach and bowels, as it was latterly when the patient became sensible of her danger, the result might have been very different. That Mrs. Lawson's second illness would have terminated in the same way as the preceding case, but for the happy influence of the medicine in question, I have not a doubt.

On the 5th of June, 1820, I was called to a young woman, aged nineteen, who had been seized, at six o'clock in the morning, with violent pain in the bowels. She had been five hours ill before I saw her, and her whole body was in a violent tremor from excess of suffering. Pulse 100, and skin hot. I bled her to twenty-five ounces, with great relief: the tremor left her, and she could lie quiet. This done, I ordered a fourth of a grain of emetic tartar, to be repeated every hour till I saw her again. No sensible effect was produced by the two first doses; but, soon after taking the third, she vomited copiously and easily; and very shortly after fell asleep, and slept for eight hours uninterruptedly. I was called at twelve at night, and found the pain and tremor had returned with as much violence as ever. No more of the medicine had been taken after the third dose, although I had directed it to be resumed every two hours, upon her awaking from sleep. I bled her again to twenty-five ounces, and with the same relief as before. Next day, the pain was only slightly felt. I ordered her a mild aperient, and to resume the antimonial. As the pain was not urgent, the latter part of the directions was not attended to. On the succeeding day, however, it again became serious, when the patient very willingly took half a grain of the emetic tartar in solution. At this time she was sitting at the fire, on a chair, which she preferred to lying in bed. Soon after taking the medicine, she fainted three several times, but without feeling distress of any kind. I was sent for on the occasion, and found my patient laughing at her friends for being alarmed. This completed the cure: the pain never returned.

Two days after, her father, who lived six miles off, came to take her home, till she recovered strength: but so convinced was she the emetic tartar contributed mainly to the cure, that she insisted on carrying a phial of antimonial wine along with her, with the determination of taking it as she did the last dose, in the event of a relapse. It may be reckoned a rough sort of remedy which produces fainting three several times, and at short intervals; but the professional reader will readily admit that, in the present case, this effect was to be attributed as much to the loss of blood and the erect posture, as to the medicine. Every body knows it is no uncommon thing for persons to faint upon taking the erect posture after being liberally bled, though they may have taken no medicine whatever.

In the course of last winter, one of my servants, nineteen years of age, had repeated attacks of an inflammatory nature, which uniformly yielded to emetic tartar. Early in the spring, she was suddenly seized with symptoms of what I considered inflammatory fever,—such as oppressive head-ache, hot skin, urgent thirst, a frequent and hard pulse, beating 130 strokes in a minute.

In the course of three days, I bled her twice in the arm, ordered her brisk purgatives, and administered abundance of emetic tartar; every dose of which I took care should produce nausea, or a state approaching to it. I carried the medicine farther, indeed, than I could otherwise have done, by giving the patient plenty of very brisk small-beer, in which she delighted, and by making her eat all the foam before she came to the fluid. By these means her stomach was kept perpetually full of carbonic acid gas, which prevented the nausea which would otherwise have occurred. Considering the measures I adopted as pretty active, I was astonished I never could bring the action of the system below 110 strokes of the pulse in a minute: I suspected, therefore, there was something peculiar in the case; but never once dreamed I was contending with a disease which, without doubt, my practice greatly modified, but could not prevent running its course. On the morning of the fourth day, she was sent to the Royal Infirmary, and small-pox appeared in the evening. The eruption proved scanty, and the disease altogether mild. She had been vaccinated when young, and there was still a considerable pit at the place of inoculation.

Some weeks afterwards, and when she had completely recovered, this girl was suddenly seized, one morning, with most violent pain in her breast, as she described it. She had been ill an hour before I saw her, and was then in a most pitiable condition. From the suddenness and violence of the attack, combined with the position she assumed during a paroxysm, I concluded the breast could not be the seat of the complaint. Desiring her, therefore, to put my hand to the part affected, she placed it directly on the stomach. She had already drank warm water, and applied warm flannel, without effect. Although I

had often observed that a very minute quantity of emetic tartar nauseated this patient, and that thirty or forty drops of antimonial wine produced distressing sickness, *when first administered,* I gave her, on the present occasion, double that dose, or eighty drops; and ordered her to keep her bed. I determined to remain at home to watch the effects of the practice; but in a quarter of an hour was informed, that "Betty was at her work in the kitchen!" No other sensible effect did the medicine produce than immediate, complete, and permanent relief from pain.

*Edinburgh; 5th January,* 1824.

